# Denosumab for treating periprosthetic osteolysis: a feasibility study

**DOI:** 10.1186/s13104-025-07216-0

**Published:** 2025-04-08

**Authors:** Michael Axenhus, Henrik Bodén, Paula Kelly-Pettersson, Olof Sköldenberg

**Affiliations:** 1 Department of Clinical Sciences at Danderyd Hospital, Karolinska Institutet, Stockholm, Sweden; 2 Department of Orthopaedics, Danderyd University Hospital, Entrévägen 2 182 68, Danderyd, Stockholm, Sweden

**Keywords:** Total hip arthroplasty, Osteolysis, Denosumab, Randomized controlled trial, Outcome, Computed tomography

## Abstract

**Objective:**

Wear-induced osteolysis is a leading cause of late failure in total hip arthroplasty (THA). Denosumab, a RANKL inhibitor, suppresses osteoclast activity and may slow osteolytic progression. This feasibility study aimed to assess the practicality of conducting a randomized, double-blind, placebo-controlled trial evaluating Denosumab’s effect on periprosthetic osteolysis in asymptomatic THA patients.

**Results:**

Twelve patients were enrolled; ten completed follow-up. No significant difference in lesion volume change was observed between groups (Denosumab: +1.53 cm³; Placebo: +0.49 cm³). Secondary clinical outcomes also showed no notable differences. The trial protocol, recruitment, treatment, and follow-up were feasible, though slow enrollment limited statistical power. This study demonstrates the feasibility of a larger trial investigating Denosumab for osteolysis prevention.

**Trial registration:**

Clinicaltrails.gov, NCT02299817. Registered 20 November 2014. https://www.clinicaltrials.gov/study/NCT02299817?term=Denosumab%20for%20Treating%20Periprosthetic%20Osteolysis.%26;rank=1

**Supplementary Information:**

The online version contains supplementary material available at 10.1186/s13104-025-07216-0.

## Introduction

Total hip arthroplasty (THA) is one of the most cost-effective and quality of life restoring surgical procedures in orthopaedics and more than 2 million patients undergo THA worldwide annually [1, 2]. Although THA generally leads to remarkably good outcomes, more than 100, 000 patients each year have to undergo a risk-filled and costly revision surgery due to aseptic loosening caused by osteolysis. This cell-mediated inflammatory response to wear debris from the artificial joint is one of the major factors in reducing the longevity of a THA [3–5]. The risk of failure is highest in younger males with a 30% risk for revision surgery within 10 years [6, 7]. Despite continual changes in surgical technique and implant design, the revision THA burden has not decreased over time and is currently around 10% in Sweden and 17% in the US [8, 9].

Osteolytic lesions around well-fixed orthopaedic implants are notoriously difficult to detect and are, 7–14 years after surgery, present in 10–70% of hips [10, 11]. They are in almost all cases asymptomatic and can only be detected with a reasonably good sensitivity and specificity using computed tomography (CT) or magnetic resonance imaging (MRI) [12]. The lesions typically occur more than 5 years after surgery and, when extensive, undermine the bony fixation of the implant thereby leading to loosening of the artificial joint [11]. There is little data on the development and progression of osteolysis around hip implants and there are few studies where osteolytic lesions have been systematically followed over a number of years using CT or MRI [13, 14]. Denosumab has however, to the best of our knowledge, not been used to try to prevent the progression of osteolysis and aseptic loosening in THA. We hypothesized that Denosumab is effective in reducing wear-induced osteolysis around uncemented acetabular implants in THA.

## Methods/Design

### Study design

A randomized, double-blind, placebo-controlled trial was conducted at the Orthopaedic Department of Danderyd University Hospital in collaboration with the Karolinska Institutet in 2015–2021. Patients were randomized in a 1:1 ratio to placebo or Denosumab. Osteolytic lesion volume (< 10 cm³/≥10 cm³) at screening and physical activity according to Johnston (< 4-low activity level/≥4-high activity level) were used as stratification to ensure that these are evenly distributed between the two groups [14–16]. The Ethics Committee of the Karolinska Institute and the Swedish Medical Products Agency approved the study. The trial was initiated, designed, and performed as an academic investigation and is registered at ClinicalTrials.gov (NCT02299817). The guidelines of the CONSORT Statement were followed [17]. All study data was collected and managed in a digital Case Report Form (CRF) using REDCap electronic data capture tools hosted at Karolinska Institutet [18].

### Study population

We included patients, aged 40–85 years, with a primary THA performed due to osteoarthritis or congenital dysplasia of the hip ≥ 7 years before inclusion and who have an osteolytic lesion of at least 4 cm³ and at most 40 cm³ around an uncemented acetabular component with a polyethylene liner. Exclusion criteria included pain from the hip (Visual analogue score ≥ 3), any surgery of the hip after index operation, any previous use of bisphosphonates and inflammatory arthritis. A detailed inclusion and exclusion list is provided (Table 1).

**Table 1:** Detailed inclusion and exclusion criteria

Detailed inclusion and exclusion criteria
**Inclusion criteria**
o Age 40–85 years
o Short Portable Mental Status Questionnaire (SPMSQ) also named Pfeiffertest ≥7
o Male and females
o The primary THA performed between 7 to 20 years before inclusion.
o The primary THA performed due to osteoarthritis or congenital dysplasia of the hip.
o Uncemented cup fixation
o Baseline osteolytic lesion of at least 4 cm³ and at most 40 cm³ around an uncemented acetabular component with a polyethylene liner.
o Participant is willing and able to follow study protocol and has provided informed consent prior to any study specific procedures.
**Exclusion criteria**
o For women of childbearing potential: Subject refuses to use 1 highly effective method of contraception (contraceptive pill, intra uterine contraceptive device) for the duration of the study and for 10 months after the last dose of study medication.
o For males with a partner of childbearing potential: Subject refuses to use 1 highly effective method of contraception for the duration of the study and for 10 months after the last dose of study medication.
o For males with a partner who is pregnant: Subject refuses to use a condom for the duration of the study and for 10 months after the last dose of study medication.
o Pain in the operated hip (because the presence of hip pain in combination with an osteolytic lesion is an indication for revision surgery). VAS > 3
o Previous revision surgery of the hip i.e. exchange of any inplant after the primary surgery
o Inflammatory arthritis
o Previous participation in clinical trials with Denosumab or administration of commercial Denosumab (Prolia™ or Xgeva™)
o Currently enrolled in or has not yet completed at least 1 month since ending other investigational device or drug trial(s), or subject is receiving other investigational agent(s).
o Treatment with any intravenous bisphosphonate, fluoride (except for dental treatment) or strontium ranelate within 5 years prior to inclusion.
o Treatment with any oral bisphosphonate within 1 year prior to inclusion.
o Treatment with cortisol or cytostatic drugs within 6 months prior to inclusion.
o Administration of any of the following treatments 3 months prior to screening:
• Anabolic steroids or testosterone
• Glucocorticosteroids (≥ 5 mg prednisone equivalent per day for more than 10 days or a total cumulative dose of ≥ 50 mg)
• Calcitonin
• Calcitriol or vitamin D derivatives [vitamin D contained in supplements or multivitamins is allowed]
• Other bone active drugs including anti-convulsives (except benzodiazepines) and heparin
• Chronic systemic ketoconazole, ACTH (adrenocorticotrophic hormone), cinacalcet, aluminum, lithium, protease inhibitors, methotrexate, gonadotropin-releasing hormone agonists.
• Androgen deprivation therapy
o Hypocalcaemia.
o Bone metabolic disorders (such as OI, PHPT, Paget)
o History of osteonecrosis of the jaw and/or recent tooth extraction or dental surgery; or planned invasive dental proceedures during the study
o Serum 25-OH D < 20 ng/ml
o Significant malabsorption including Celiac Disease, Short Bowel Syndrome, Crohn’s Disease, Previous Gastric Bypass.
o Active cancer and/or malignancy in last 5 years (except cervical carcinoma in situ or basal cell carcinoma)
o History of solid organ or bone marrow transplant.
o Hypersensitivity to any components of study drug.
o Intolerance to calcium supplements.
o Pregnancy and/or currently lactating.
o Significantly impaired renal function as determined by a derived glomerular filtration rate (GFR) using Cockcroft Gault formula of ≤ 30 mL/min/1.73 m2
o Elevated transaminases ≥ 2.0 x upper limit of normal (ULN); Elevated total bilirubin (TBL) > 1.5 x ULN.

### Treatments

Half of the patients received a 1 ml subcutaneous injection of Denosumab 60 mg on day one and every 6 months with last treatment at 30 months. The other half received placebo injections. Calcium and Vitamin D (500 mg + 400 IE) was administrated twice daily from day 1 to 3 years to all patients.

### Endpoints

The primary endpoint variable was the change in volume of the osteolytic lesion over 3 years (measured with 3D-CT in cm³):

$$\:E{fficacy}_{3\:years}={Volume}_{3\:years}-{Volume}_{baseline}$$.

Secondary endpoints included change in volume of the osteolytic lesion over 2 years and clinical outcome scores. The Harris hip score (HHS) was used to assess patient-reported functional hip status and physical activity [19, 20]. Health-related quality of life was assessed by the EQ-5D (EuroQoL) [21].

### Osteolysis

We used a high-resolution 3D-CT at inclusion to detect and measure the volume of the osteolysis according to Howie et al. [13, 14]. The scan was repeated at 2 and 3 years. Osteolysis is defined as a demarcated nonlinear osteolytic lesion > 3 mm. The measurements were performed by a technician otherwise not involved in the study and blinded to treatment and who is trained in quantitative CT analysis. 3D-CT has been shown to have an 80% sensitivity and a 100% specificity in detecting osteolytic lesions around uncemented acetabular components [22]. Once detected, the volume of the lesion can be measured with an error of mean (SD) 7.1% ±24.1% (0.3 ± 1.1 cm³) [22].

### Adverse events

Adverse events (AEs) were defined as any untoward medical occurrence in a patient or clinical investigation subject administered a pharmaceutical product and that does not necessarily have a causal relationship with this treatment (Table 2). The principal investigator would record and grade all adverse events according to certain criteria (Supplemental Table 1).

**Table 2:** Clinical outcomes at screening, 2 and 3 years follow up

	Denosumab	Placebo	*p*-value
**EQ-5D**			
Screening	69,6 ± 17,7	83,6 ± 9,3	0.14
2 years	72,0 ± 16,0	85,8 ± 12,3	0.21
3 years	72,8 ± 18,3	80 ± 17,7	0.58
**HHS**			
Screening	91,6 ± 9,6	90,4 ± 8,1	0.82
2 years	87,6 ± 10,2	78,8 ± 15,2	0.37
3 years	82,6 ± 16,6	88,4 ± 6,6	0.53

### Sample size

In a pilot study using 3D-CT Schwarz et al. identified 19 patients with osteolytic lesions around an uncemented acetabular cup used in THA [23]. After 1 year the volume of the lesions had increased with mean (SD) 3.19 (3.67) cm³. Howie et al. studied the natural progression of osteolytic lesions after THA with 3D-CT [14]. They scanned 30 patients with a known osteolytic lesion 15 months (range, 12–27) after the initial scan and found that 16 (53%) of the lesions had increased in volume. The lesions most likely to increase in size was ≥ 10 cm³ at the initial scan. The median volume increase was 3 cm³ during the 15 months studied. Based on the work by Schwarz and Howie and thereby assuming a 3 cm³ increase annually and a 3-year study period would indicate that we are looking for a mean increase of 9 cm³ with a SD of 8 cm³. The SD is estimated by dividing Howie et al.´s range of lesion size by 4 as suggested by Hozo et al. [24]. For Denosumab we assume that it will reduce the progression of osteolysis about 50% compared to placebo. Patients treated with Denosumab would therefore have a mean increase of 4.5 cm³ (0.5 × 9 cm³) after 3 years. A two-tailed superiority sample size calculation for the primary endpoint variable change in osteolytic volume after 3 years, assuming a progression of volume of 9 cm³ for the placebo and 4.5 cm³ for the Denosumab group and with a SD of 8 cm³ in both groups and a p-value of 0.05 means 50 subjects in each group for an 80% power. We aimed to include 55 patients in each group to allow for loss to follow-up and loss of data.

### Statistics

The analyses were performed on the basis of the intention-to-treat principle. Unpaired Student’s t-test was used for comparison of change in osteolysis volume at 2 and 3 years. Descriptive statistics (means and standard deviations) was used to describe patient characteristics and outcome variables at the measurement points. We used non-parametric tests for HHS and EQ-5D. For subjects that withdrew from the study before year 3, the data from the last observation was carried forward.

## Results

**Fig. 1:**
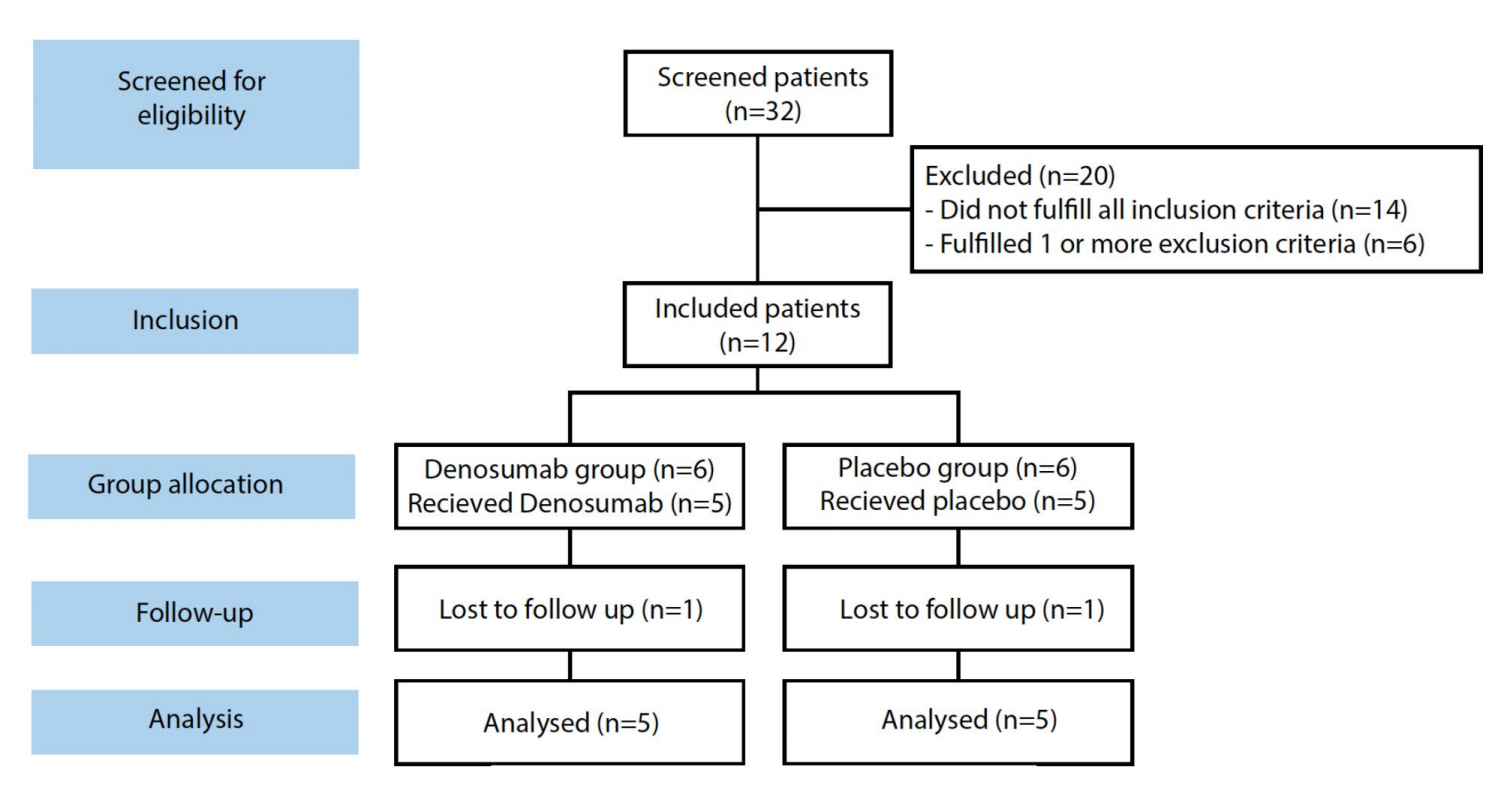
CONSORT flow diagram

We screened a total of 32 patients of which 12 were included. We lost 1 patient in each group during follow-up and analysed 5 in each group (Fig. 1). Characteristics were similar between groups (Table 3).

**Table 3:** Descriptive characteristics of the included study participants

				Denosumab				Placebo			
				**Mean**	**SD**	**N**	**%**	**Mean**	**SD**	**N**	**%**
Age				77	5			70	8		
Sex											
	Male					3	60			4	80
	Female					2	40			1	20
Height (cm)				172	12			178	10		
Weight (kg)				88	15			92	27		
Time after surgery (years)				22				19	4		
ASA	1					0	0			2	40
	2					5	100			0	0
	3					0	0			3	60
Volume osteolysis (cm3)				8.1	5.8			5.4	6.5		

### Primary endpoint

**Fig. 2:**
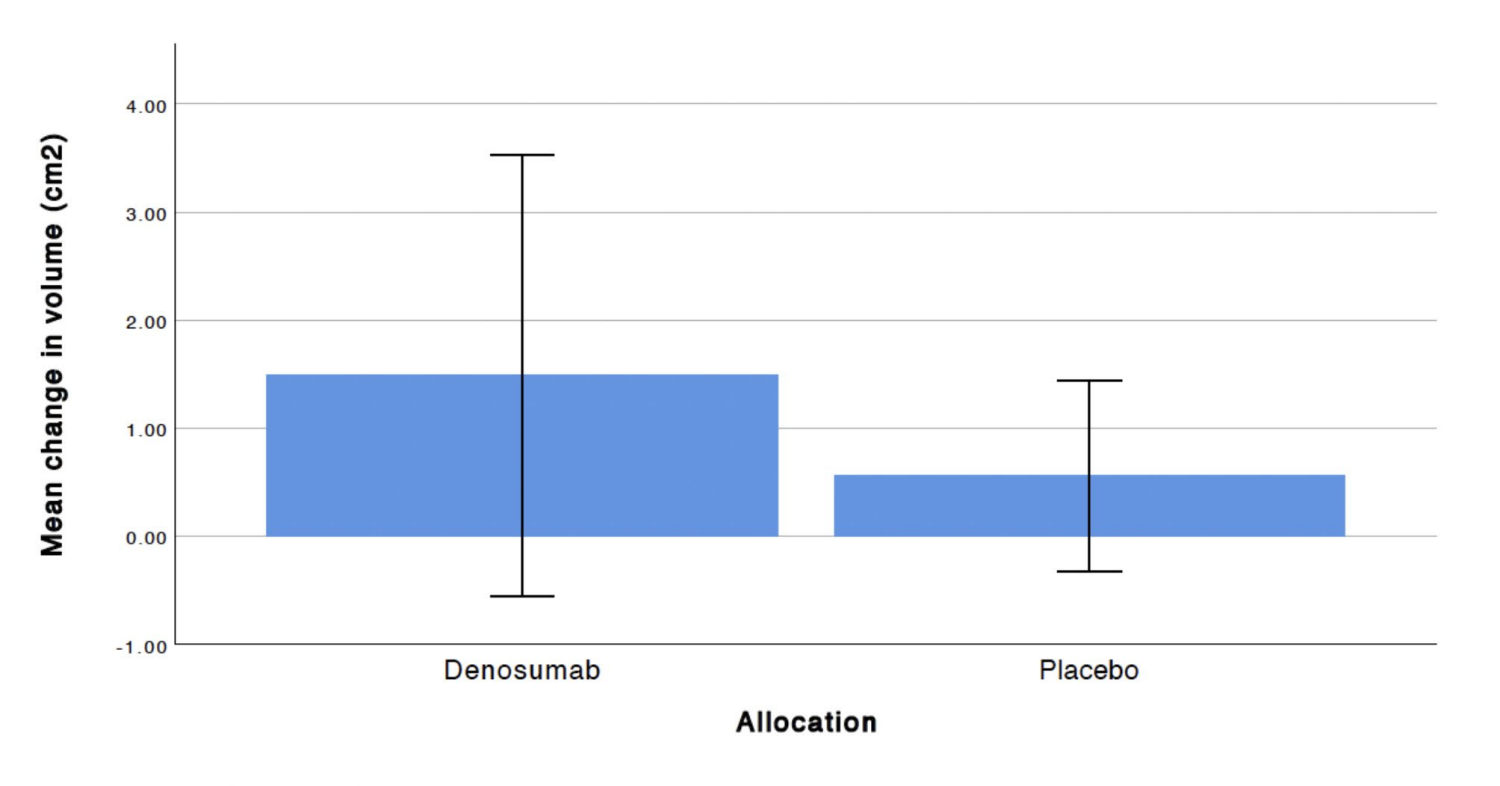
Mean volume change in osteolytic lesions at 3 years follow up. Error bars indicate 95% CI

The change in volume of the osteolytic lesion as measured by 3D-CT over 3 years showed a larger change in volume in the Denosumb group compared to the placebo group, but there was no significant difference between groups. The mean change in volume was 1,53 cm^2^ (CI -0.51 to 3.56) for the Denosumab and 0,49 cm^2^ (CI -0.22 to 1.24) for the placebo group respectively (Fig. 2).

### Secondary endpoint

**Fig. 3:**
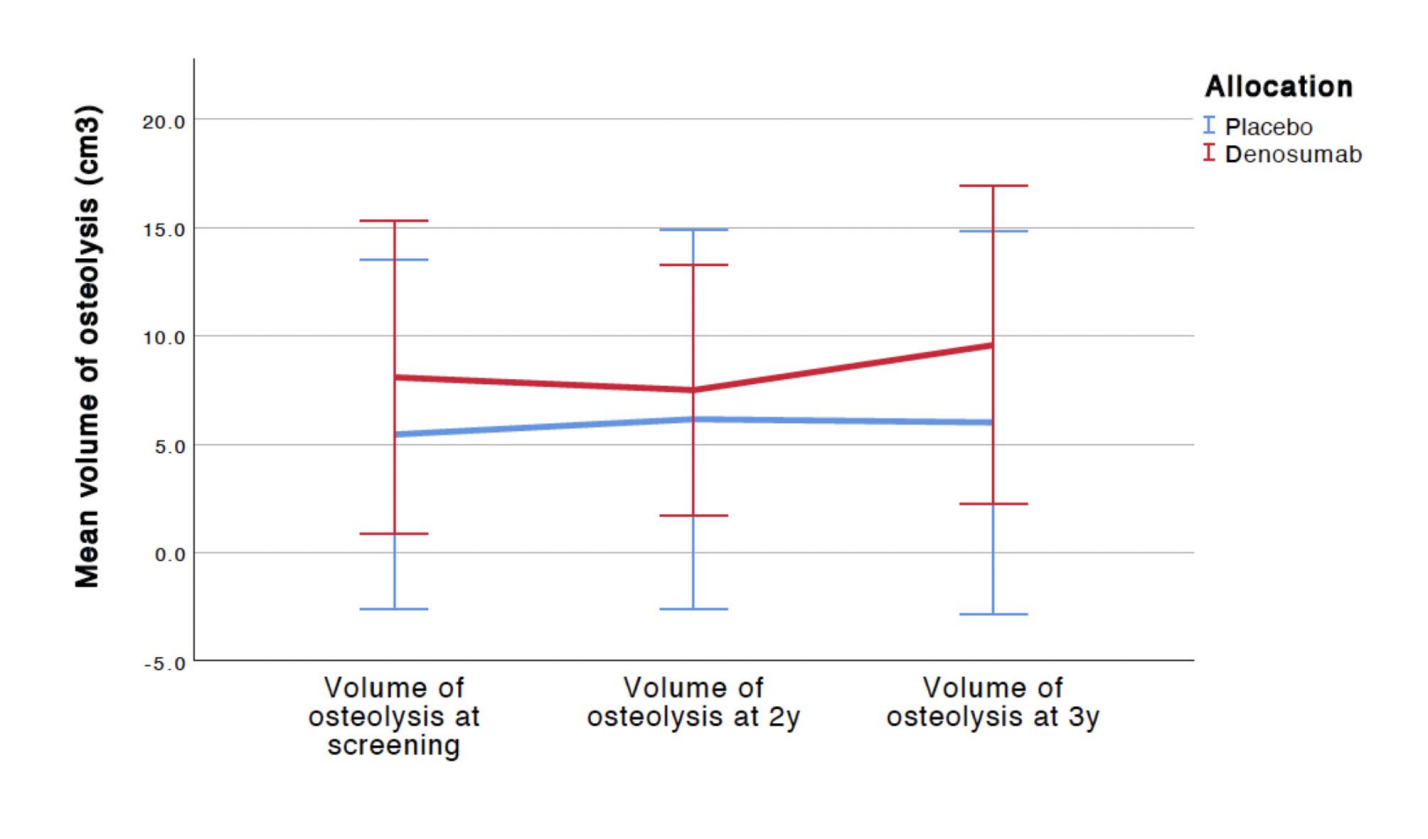
Mean volume change of osteolysis lesions during 2 and 3 years follow up

There was no significant difference in the volume of the osteolytic lesions at the 2 years and 3 years follow up (Fig. 3). There was no significant difference between clinical outcomes at 2 and 3 years follow-up (Table 2). No AE occurred in either group. There were no signs of the initial osteolysis rate influencing patient outcomes.

## Discussion

This trial demonstrates that a full-scale RCT investigating Denosumab for periprosthetic osteolysis is feasible but was challenged in our case by recruitment barriers and the strict criteria. Although underpowered, our study establishes the groundwork for larger trials and supports further investigation of Denosumab in this context.

We included patients with asymptomatically osteolytic lesions. These lesions are, based on the current literature, highly likely to progress over the years and lead to massive osteolysis and require revision surgery. The risk of dislocation and deep periprosthetic joint infection is higher following revision arthroplasty than after a primary THA [25]. The clinical outcome regarding hip function is also poorer after revision surgery [26].

The susceptibility to develop osteolysis has been shown to vary between individuals [27, 28]. It should be noted that bisphosphonates have been found to be effective in reducing disuse bone atrophy (a.k.a. “stress-shielding”) around orthopaedic implants but have not been effective in preventing progress of osteolytic lesions [29, 30]. Inhibitors of cathepsin K are in development for treatment of osteoporosis and have recently been shown to reduce fracture risk in patients with osteoporosis [31]. Recently, Denosumab was found to be effective in preventing osteoporosis related fractures in post-menopausal women by blocking RANKL and thereby inhibiting the development and activity of osteoclasts [32]. In a recently published animal model of prosthetic loosening, targeting osteoclast recruitment via RANKL inhibition was found to be effective in targeting osteoclasts [33]. Taken together, these findings indicate a potential for Denosumab to inhibit osteolysis on a biochemical level and future studies are still warranted in order to elucidate this hypothesis. There are a few reported cases of atypical femoral fractures after Denosumab treatment, although these tend to be rare and often associated with long term oral bisphosphate treatment [34, 35].

In conclusion, our study did not meet its primary outcome, likely due to stringent inclusion criteria and a smaller-than-planned sample size. We did find that the volume of osteolysis induced by Denosumab was greater than anticipated. This could indicate that Denosumab might have an inductive effect on osteolysis, and future studies should consider this endpoint.

Despite its limitations, this study represents the first clinical exploration of Denosumab’s potential role in managing periprosthetic osteolysis. While definitive conclusions cannot be drawn, the findings offer valuable preliminary data to inform future trial design. Given Denosumab’s well-established mechanism of inhibiting osteoclast activity via RANKL suppression, further investigation remains scientifically justified. Continued research is warranted, especially considering the clinical burden and risks associated with revision hip arthroplasty.

### Limitations

This study’s primary limitation is its small sample size of 12 participants, reducing statistical power and limiting generalizability. Future trials are encouraged to use broader inclusion criteria in order to reach statistical power. A multi-center design would be preferable.

The three-year follow-up may not capture long-term outcomes such as progression to revision surgery. Additionally, the use of 3D-CT, while specific, may have limited accuracy in detecting small changes in osteolytic lesion volume. Researchers might explore alternative imaging or biomarkers for osteolysis progression.

Lastly, the study’s focus on older adults with long-standing hip replacements restricts the generalizability of findings to other patient groups. Larger and more diverse populations are required in order to generalize findings.

## Electronic supplementary material

Below is the link to the electronic supplementary material.

Supplementary Material 1: Title of data: Supplemental Table 1. Description of data: Definition of safety assessment and descriptions.

## Data Availability

Data is available from the corresponding author on reasonable request.

## Abbreviations

3D-CT: Three-Dimensional Computed Tomography
AE: Adverse Event
ALF: Agreement on Medical Training and Clinical Research
ASA: American Society of Anesthesiologists
CI: Confidence Interval
CONSORT: Consolidated Standards of Reporting Trials
CRF: Case Report Form
EQ-5D: EuroQol-5 Dimension
HHS: Harris Hip Score
IL: Interleukin
IP: Interferon-Inducible Protein
MCP: Monocyte Chemoattractant Protein
MRI: Magnetic Resonance Imaging
RANK: Receptor Activator of Nuclear Factor Kappa B
RANKL: Receptor Activator of Nuclear Factor Kappa B Ligand
REDCap: Research Electronic Data Capture
SD: Standard Deviation
THA: Total Hip Arthroplasty
